# The mRNA Binding Proteome of Proliferating and Differentiated Muscle Cells

**DOI:** 10.1016/j.gpb.2020.06.004

**Published:** 2020-12-16

**Authors:** Monika Hiller, Miriam Geissler, George Janssen, Peter van Veelen, Annemieke Aartsma-Rus, Pietro Spitali

**Affiliations:** 1 Department of Human Genetics, Leiden University Medical Center, Leiden 2333 ZC, the Netherlands; 2 Center for Proteomics and Metabolomics, Leiden University Medical Center, Leiden 2333 ZC, the Netherlands

**Keywords:** RNA binding protein, Interactome, Duchenne muscular dystrophy, Proteomics, Skeletal muscle

## Abstract

Muscle formation is a coordinated process driven by extensive gene expression changes where single cells fuse together to form multinucleated muscle fibers. Newly synthesized mRNAs are then regulated by **RNA binding proteins** (RBPs), affecting post-transcriptional transcript metabolism. Here, we determined how large-scale gene expression changes affect the catalog of RBPs by studying proliferating and differentiated muscle cells in healthy and dystrophic conditions. Transcriptomic analysis showed that the expression of more than 7000 genes was affected during myogenesis. We identified 769 RBPs, of which 294 were muscle-specific and 49 were uniquely shared with cardiomyocytes. A subset of 32 RBPs (half of which were muscle-specific) was found to be preferentially associated with target mRNAs in either myoblasts (MBs) or myotubes (MTs). A large proportion of catalytic proteins were bound to mRNAs even though they lack classical RNA binding domains. Finally, we showed how the identification of cell-specific RBPs enabled the identification of biomarkers that can separate healthy individuals from dystrophic patients. Our data show how **interactome** data can shed light on new basic RNA biology as well as provide cell-specific data that can be used for diagnostic purposes.

## Introduction

Skeletal muscle development and regeneration require a coordinated series of events during which single proliferating muscle stem cells fuse to form multinucleated muscle fibers. This unique process is driven by a cascade of transcriptional events that determine whether cells are committed to become contracting muscle or remain in a quiescent state. While transcriptional studies have elucidated the gene expression changes required for successful muscle formation in *in vitro* and *in vivo* models [1], [2], [3], studies focusing on how the observed gene expression changes are regulated by RNA binding proteins (RBPs) are lacking. RBPs are necessary for mRNAs to exert their functions within the cell environment by forming ribonucleoprotein complexes. RBPs have been described to affect the way mRNAs and miRNAs act by coordinating mRNA turnover, localization, and translation [4], [5], [6], [7], [8]. The fundamental role of RBPs is further underlined by their role in the development of genetic conditions such as neurodegenerative diseases [9] and neuromuscular diseases, for example in myotonic dystrophy type 1, which is caused by the sequestration of specific RBPs [10], [11].

In the last five years, there has been great progress in the identification of RBPs. The development of improved enabling technologies has allowed high-throughput capture and identification of RBPs bound to polyadenylated RNAs. One of these methods is the interactome capture method, which has been used to identify the repertoire of RBPs in a variety of human and murine cell lines [12], [13], [14], [15], [16], [17]. These studies have not only expanded our knowledge of the hundreds of proteins able to bind RNA, but they have also shown how several proteins bind their RNA targets through unconventional RNA binding domains [18]. Interestingly, different cell types have shown different repertoires of RBPs, suggesting that particular proteins may act as RNA binders in certain cell types but not in others.

In this work we aimed to uncover the catalog of RBPs present in proliferating and differentiated muscle cells and to study whether differences exist in cells obtained from patients affected by Duchenne muscular dystrophy (DMD), where the gene expression signature is highly altered.

## Results

### Interactome capture enables identification of RBPs in muscle cells

**Figure 1:**
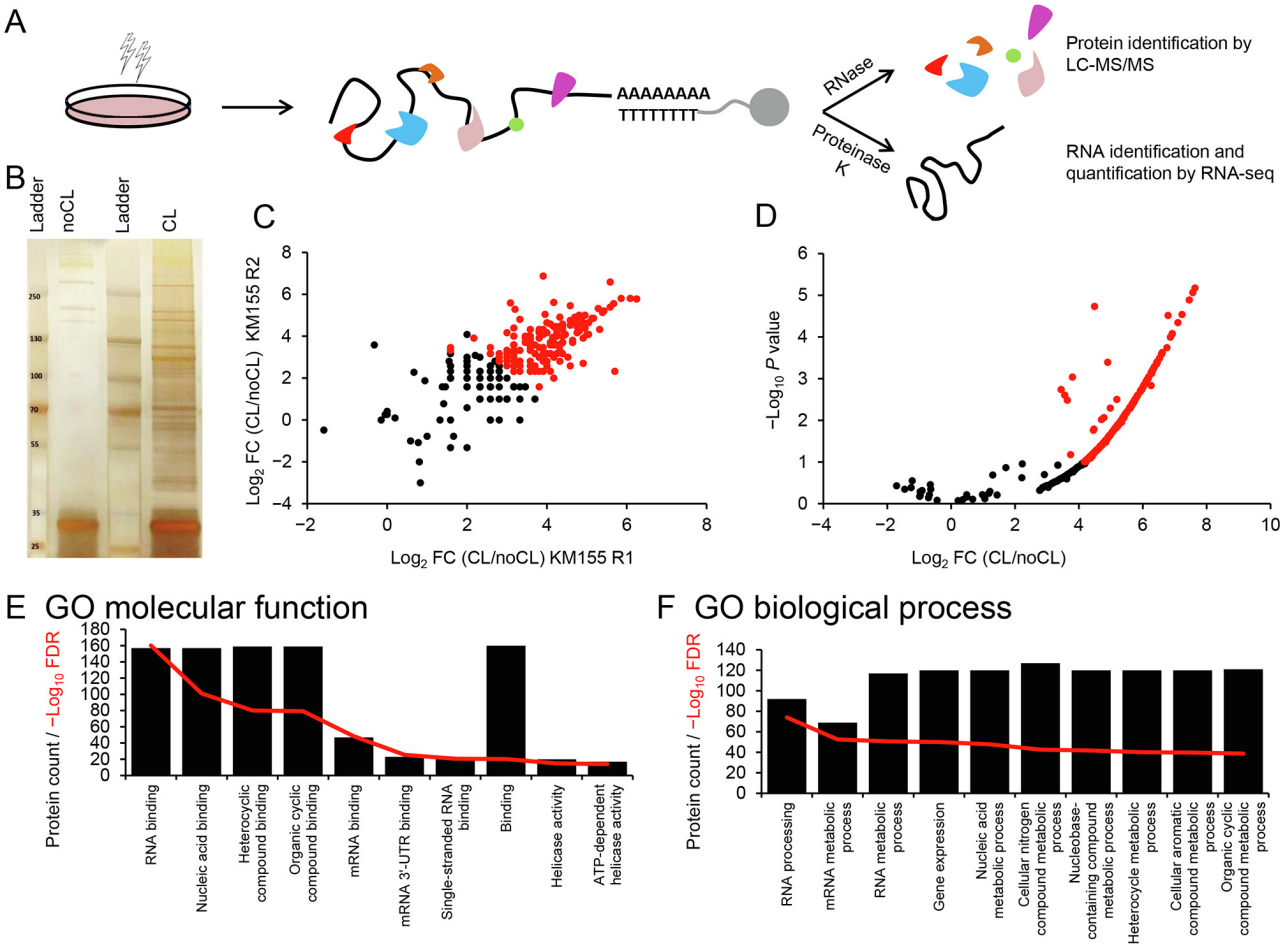
**Interactome capture shows enrichment in RBPs in muscle cells** **A.** Schematic representation of the procedure including UV crosslinking of cells, poly-A mRNA capture, and either RNase or proteinase K digestion to analyze proteins and RNA, respectively. **B.** Representative image of silver staining of SDS-PAGE gel. **C.** Scatter plot showing the log_2_ ratios between peptide counts +1 in a CL *vs*. noCL sample of two independent replicates both obtained from HC KM155 immortalized MB cells. Red dots represent proteins that show a positive FC in both CL *vs*. noCL samples and a significant change following differential expression analysis with DeSeq2 (adjusted *P* values by FDR < 0.1). **D.** Volcano plot showing the log_2_ FC in CL *vs*. noCL MB samples (*x*-axis) and the −log_10_*P* value (*y*-axis). A clear shift towards the right is visible, representing the enrichment of proteins in CL samples compared to noCL samples. Red dots represent proteins that show a positive FC in both CL *vs*. noCL samples and a significant change following differential expression analysis with DeSeq2. **E.** Bar graph showing the results of the GO molecular function analysis. Bar height represents the number of proteins mapped to top 10 GO molecular function terms. The red line represents the −log_10_ of the adjusted *P* values (FDR). The boundaries shown on the *y*-axis refer to both bar values and line values. **F.** Bar graph showing the results of the GO biological process analysis. Bar height represents the number of proteins mapped to top 10 GO biological process terms. The red line represents the −log_10_ of the adjusted *P* values (FDR). The boundaries shown on the *y*-axis refer to both bar values and line values. RBP, RNA-binding protein; noCL, uncrosslinked; CL, crosslinked; MB, myoblast; HC, healthy control; FC, fold change; FDR, false discovery rate.

To identify muscle-specific RBPs, proliferating muscle cells (myoblasts, MBs) were cultured and differentiated into multinucleated myotubes (MTs), mimicking the generation of muscle fibers. Both MB and MT cells were then irradiated with UV light to induce crosslinking via covalent bond formation between RNA and proteins. RBPs were purified after capturing polyadenylated mRNAs with an oligo-dT bound to magnetic beads. Samples were then split to enable both protein and RNA analyses (Figure 1A). Silver staining images showed strong enrichment in protein content in crosslinked (CL) samples compared to uncrosslinked (noCL) samples (Figure 1B, Figure S1A). To test whether the crosslinking protocol enabled protein enrichment after poly-A purification, we compared two replicates of healthy control (HC)-derived MB CL to two replicates of HC-derived MB noCL. Protein identification for both MB CL and MB noCL showed an increased number of identified proteins in CL conditions that was reproducible (Figure 1C and D). In proliferating MBs, we identified 160 RBPs characterized by a positive fold change (FC) in CL samples compared to noCL samples and a significant difference according to differential representation analysis as tested by DESeq2. DESeq2 analysis provides a formal demonstration of the enrichment; nevertheless, in this case the enrichment is also clearly visible in the gel and evident in the mass spectrometry count data (Figure 1B, Figure S1A and B). GO annotation of the identified proteins for both molecular function and biological process showed strong enrichment for RBPs and proteins involved in RNA biology (Figure 1E and F). An extended analysis involving both MB and MT samples obtained from HCs and DMD patients (a total of eight samples with four MB and four MT samples from CL or noCL conditions) was performed (data available in Table S1). This analysis again showed a significant enrichment of 124 proteins out of the identified 655 proteins in the CL samples compared to the noCL samples (Figure S1B). As expected, we were able to pull down proteins known to bind RNA such as CELF2, HNRNPM, and HDLBP, as well as RBPs involved in muscle diseases such as MBNL1, which plays a key role in the pathogenic mechanism of myotonic dystrophy type 1 (Figure S1C). Therefore, we have shown that crosslinking is required to enrich for RBPs and to study the interactome of muscle cells. Further analyses of different experimental conditions were performed by comparing CL samples only.

### Comparison of proliferating and differentiated cells

**Figure 2:**
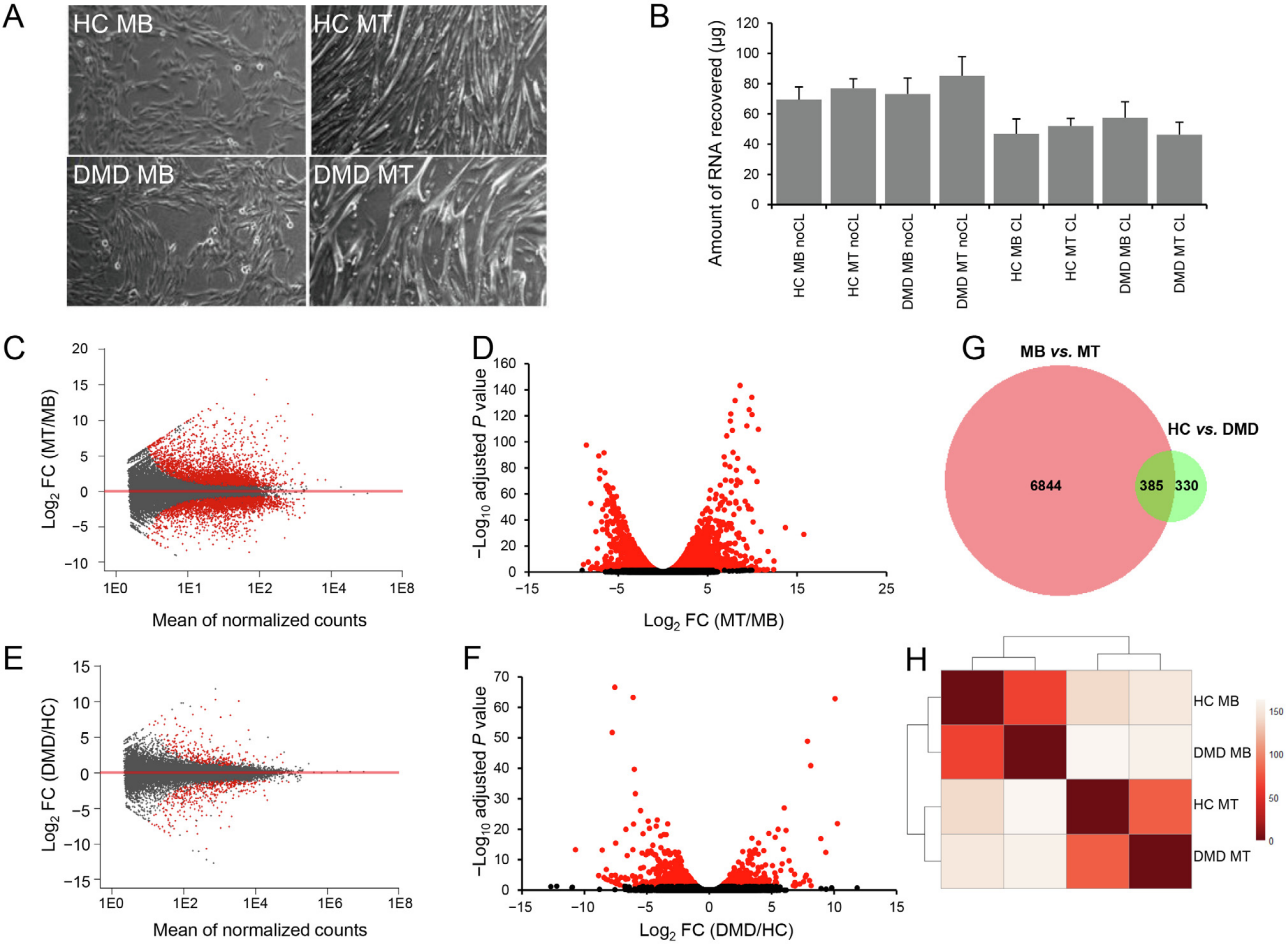
**Gene expression changes upon muscle differentiation in HC- and DMD-derived cells** **A.** Representative bright field images of proliferating MB and differentiated MT of both HC- and DMD-derived cells. **B.** Bar graph showing the amount of RNA recovered before and after crosslinking per cell type and differentiation stage (*n* = 3). A reduction of total RNA purified from CL samples is visible. Values are mean ± SD. **C.** MA plot showing gene expression changes in MT compared to MB. The *x*-axis shows the mean of normalized counts per gene, while the log_2_ of FC is plotted on the *y*-axis. Dots above the horizontal line show increased expression in MT over MB. Red dots are DEGs after multiple testing correction (adjusted *P* < 0.1). **D.** Volcano plot showing the difference in gene expression levels between MB and MT. The log_2_ FC is plotted on the *x*-axis, while the −log_10_ of the adjusted *P* value is plotted on the *y*-axis. A positive FC represents higher expression in MT compared to MB. Red dots are DEGs, while black dots are non-DEGs (adjusted *P* < 0.05). **E.** MA plot showing gene expression changes between HC- and DMD-derived cells. Dots on top of the horizontal line show increased gene expression in DMD compared to HC. Red dots are DEGs (adjusted *P* < 0.1). **F.** Volcano plot showing the gene expression changes in DMD cells. Red dots represent DEGs. Genes with positive FC represent genes with higher expression in DMD compared to HC after multiple testing correction (adjusted *P* < 0.05). **G.** Venn diagram showing the overlap in gene signature of the two comparisons. A total of 385 genes were differentially expressed between MB and MT as well as between HC and DMD. **H.** Heatmap showing gene expression data for HC MB, HC MT, DMD MB, and DMD MT. Hierarchical clustering of samples was performed to obtain distances across samples. The *dist* function was used to calculate Euclidean distances between samples based on normalized count data. The *pheatmap* R package was used to show that clustering of differentiation stage is the strongest grouping factor as shown by the color code indicating sample distances. MT, myotube; DMD, Duchenne muscular dystrophy; DEG, differentially expressed gene.

We compared proliferating single nucleated MB cells with differentiated multinucleated MT cultures from HCs or DMD patients (HC MB, HC MT, DMD MB, and DMD MT, respectively; Figure 2A). HC samples consisted of two replicates of KM155 cells and one replicate of AB117 cells, and DMD patient samples consisted of two replicates of 8036 cells and one replicate of 6311 cells (see Materials and methods for details).

#### RNA-seq analysis shows differences between cell types and between HC and DMD cells

RNA purification showed that the quantities of RNA recovered were reduced after crosslinking (Figure 2B), and the total number of reads was similar across samples (51–64 million per sample). Sequencing of the purified mRNAs was performed to assess whether the transcriptional reprogramming of cells occurred in both HC- and DMD-derived cells and to test whether differences existed between the conditions. As expected, a strong signature marked the two cell stages with over 7000 genes differentially expressed between MB and MT samples (Figure 2C and D). Overrepresentation analysis of the differentially expressed genes (DEGs) showed terms such as contractile fiber, myofibril*,* and sarcomere among the top GO terms for cellular component, indicating cellular programming towards formation of multinucleated muscle fibers (Table S2). As expected, we identified genes involved in muscle function such as those encoding dystrophin and myosin binding protein C. We also showed an increase in ribosome coverage, as previously described [16]. Pathway analysis with Reactome showed enrichment of processes involved with mRNA translation, which is compatible with the synthesis of myofibrillar proteins needed to form muscle fibers (Table S3). Analysis using WikiPathways was congruent with Reactome analysis with highlighted pathways such as EGF/EGFR signaling pathway, which induces growth, differentiation, and adhesion. About 700 genes were found to be differentially expressed between HC- and DMD-derived cells, with enrichment of GO terms such as skeletal muscle organ development and genes such as *DMD* (the affected gene, presumably undergoing nonsense mediated decay; Figure 2E and F). Pathway analysis using WikiPathways also highlighted pathways known to be affected in DMD such as oxidative stress, focal adhesion, NRF2 pathway, Wnt signaling, and TGF-β signaling (Table S4). A set of 385 common genes were found to be differentially expressed in both comparisons (Figure 2G). Remarkably, most transcriptional differences were identified between cell types but not between HC- and DMD-derived cells, indicating a conserved sequence of transcriptional events driving the development of muscle fibers even in disease, as clearly shown by clustering (Figure 2H). DEGs are listed in Tables S5 and S6.

#### *RBPs identified by mass spectrometry*

**Figure 3:**
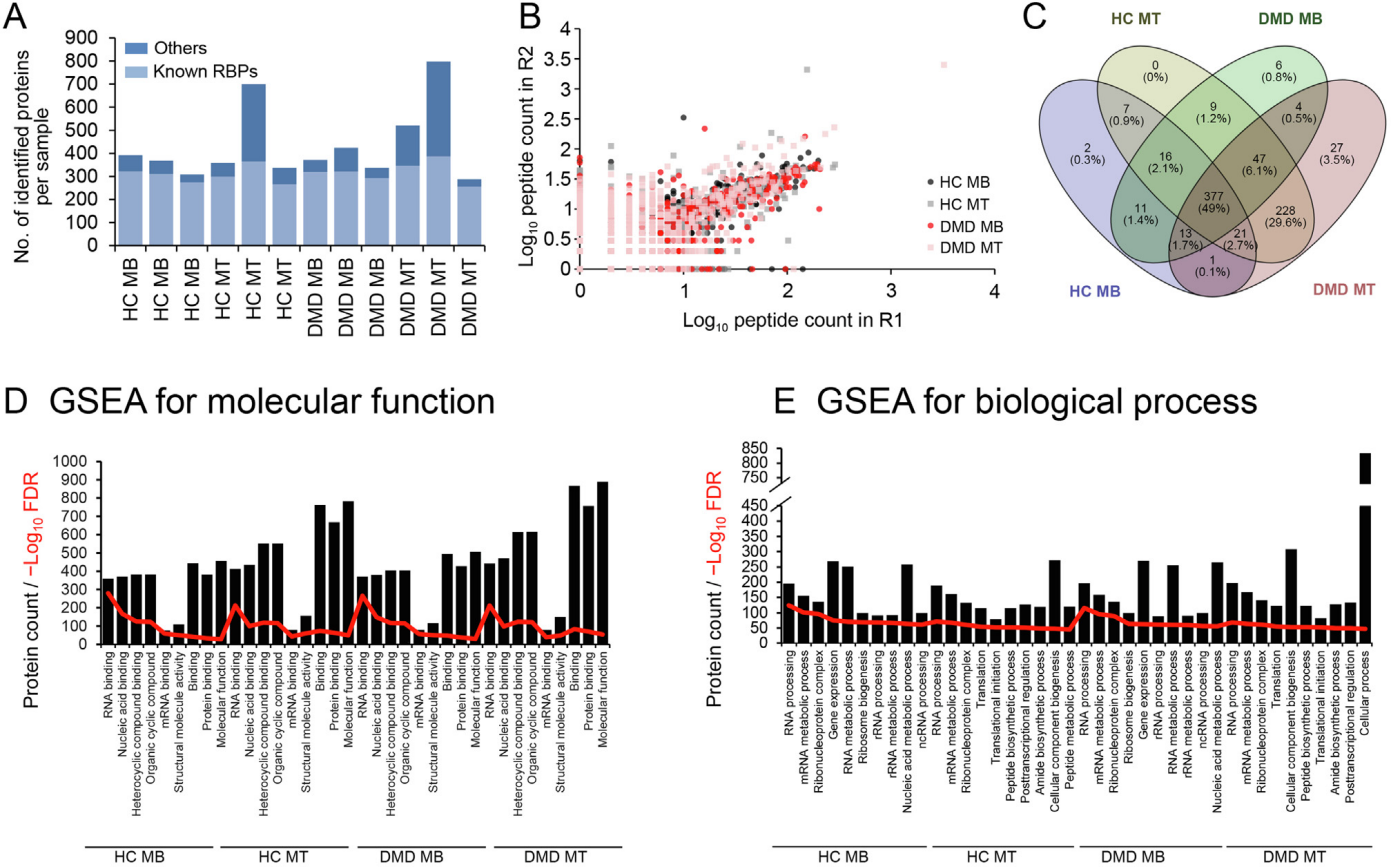
**Description of the interactome of muscle cells** **A.** Bar graph showing the number of identified proteins per sample. Light blue shows the number of proteins with known RBP properties according to GO annotation, while dark blue shows the number of other proteins identified per sample. **B.** Scatter plot showing the correlation in log_10_ of peptide counts between two replicates across four samples for 769 proteins. **C.** Venn diagram showing the overlap of identified proteins across the four different conditions. **D.** Bar graph showing the results of GSEA for molecular functions. Nine of the top 10 molecular functions were shared across the four groups (HC MB, HC MT, DMD MB, and DMD MT). Bar height shows the number of proteins mapped to the top 9 molecular functions across the groups. The red line represents the −log_10_ of the FDR. The bounds shown on the *y*-axis refer to both bar values and line values. **E.** Bar graph showing the results of GSEA for top 10 biological processes per condition. Bar height shows the number of proteins mapped to the top 10 biological processes per condition. The red line represents the −log_10_ of the FDR. The bounds shown on the *y*-axis refer to both bar values and line values. GSEA, gene-set enrichment analysis.

**Figure 4:**
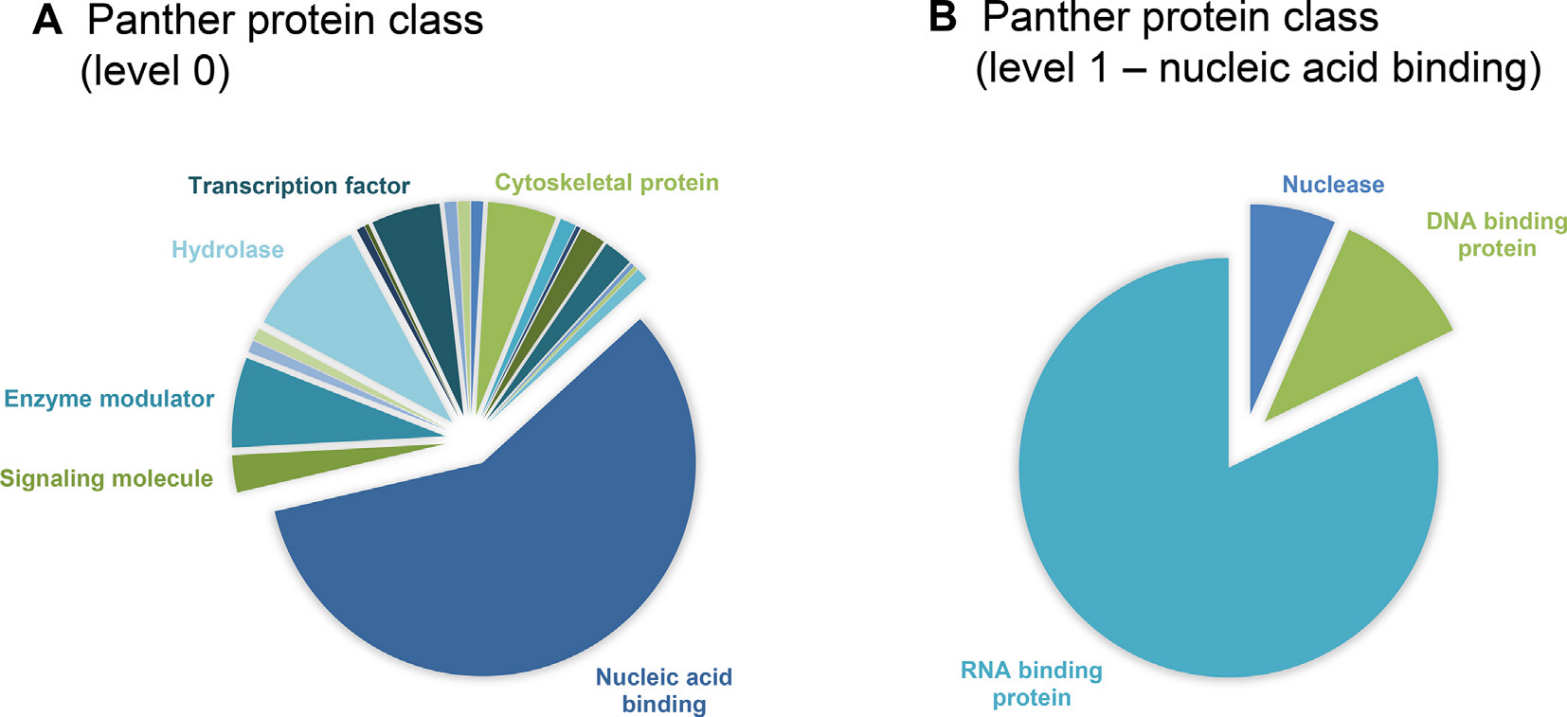
**Protein classification of HC MB shows enrichment in RBP content** Protein classification analysis was performed using Panther (http://pantherdb.org/). The list of proteins obtained from HC MB was used as example. **A.** Result of the protein classification shows clear enrichment in nucleic acid binding proteins. **B.** Pie chart showing that more than 80% of the nucleic acid binding proteins are classified as RBPs.

Analysis of proteins bound to RNA was performed on comparable amounts of proteins purified across samples and an average of 430 proteins per sample were identified (Figure 3A; Table S7). For two MT samples (one derived from a healthy subject and one from a DMD subject), a large number of non-RBPs were co-purified, which were largely composed of muscle proteins not known to be RBPs. Out of the 1135 identified proteins, a set of 769 proteins showing five or more peptide counts across samples was further analyzed. Comparison of HC MB, HC MT, DMD MB, and DMD MT replicates showed highly significant correlations in the quantities (peptide counts) of identified proteins across 769 proteins (Figure 3B, Spearman *P* ranging between 10^−142^ and 10^−9^). We then proceeded to analyze which proteins were identified in each condition by pooling together the proteins identified in the three replicates. The majority (49.0%) of RBPs were present in all cell types, with a considerable proportion (29.6%) of muscle proteins “contaminating” the MT samples (Figure 3C). Gene-set enrichment analysis (GSEA) showed that among the top 10 molecular functions per condition, nine were shared across the four groups (Figure 3D), while biological processes were more conserved within cell types (Figure 3E). Notably, all pathways point to RNA-related molecular functions. We further characterized the repertoire of identified proteins using Panther (Figure 4). Molecular function analysis mapped the majority of the proteins to three GO categories: binding, catalytic activity, and structure molecule activity (Figure S2). Sorting of proteins according to the protein classes showed that the majority of identified proteins are nucleic acid binding proteins, specifically RBPs. In this protein class we identified splicing factors (*e.g*., SUGP2), ribonucleoproteins (*e.g*., SNRPD1), and transcription and translation factors (*e.g*., FUS and PUM2). Proteins with catalytic activity were classified mostly as hydrolases (*e.g*., UPF1), transferases (*e.g*., TERT), and helicases (*e.g*., DHX9). Proteins with structure molecule activity were mostly structural components of the ribosome (*e.g*., RPS14). An overview of the protein classification per sample type is shown in Figure S2.

### Muscle differentiation affects the composition of RBPs

**Figure 5:**
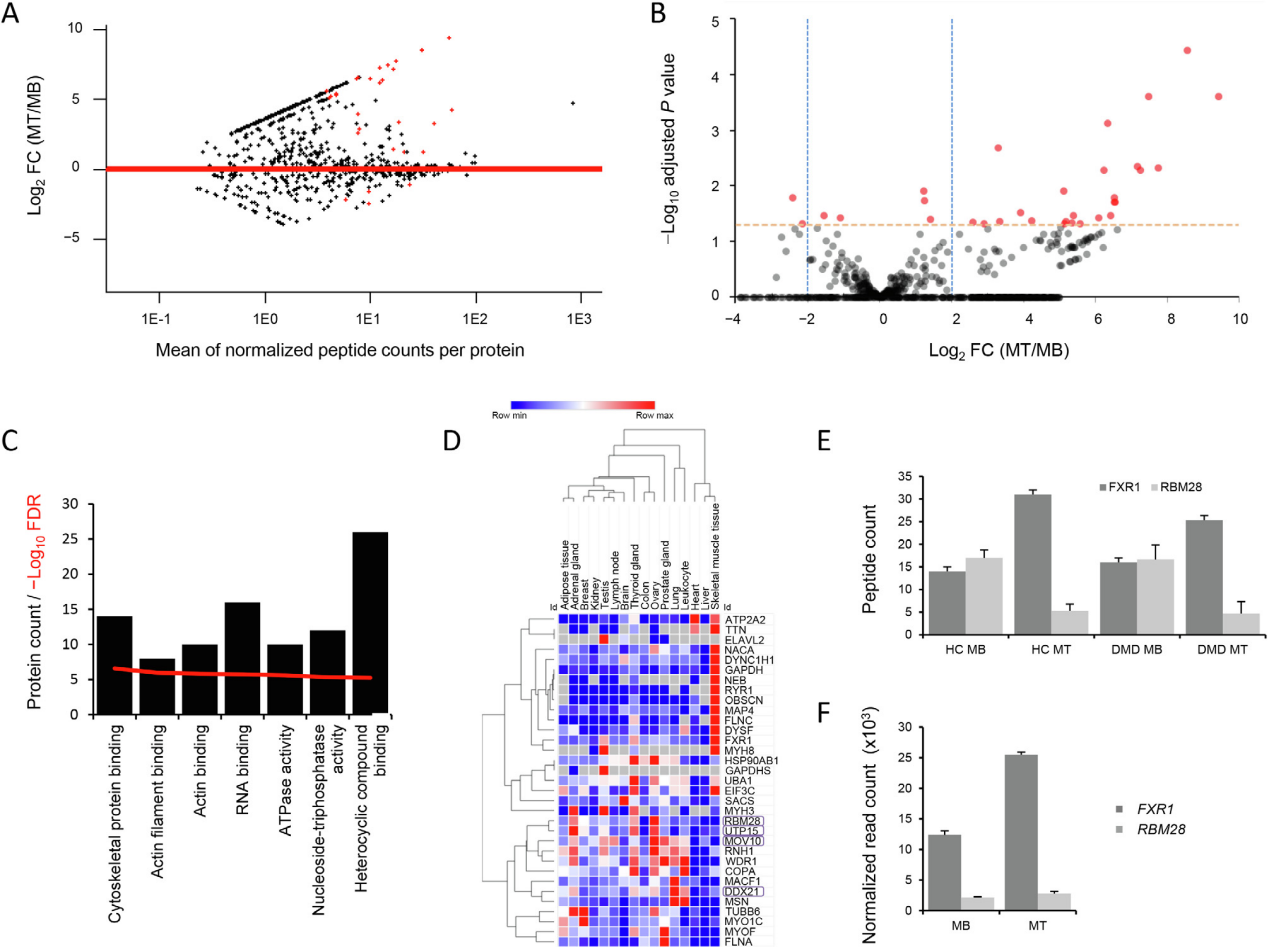
**Muscle differentiation affects the interactome** **A.** MA plot showing changes in the interactome derived peptide counts in MT compared to MB. Dots above the horizontal line show increased peptide counts in MT over MB. Red dots indicate significant differences after multiple testing correction. **B.** Volcano plot showing the differences in peptide counts between MB and MT. A positive FC represents higher peptide counts in MT compared to MB. Red dots indicate significant differences. The horizontal orange dashed line represents the 0.05 threshold of significance after multiple testing correction. Vertical blue dashed lines represent 2× FC reduction and increase. **C.** Bar graph showing the enrichment in GO molecular function terms for the differentially identified proteins. Bar height represents the number of differentially identified proteins mapped to each GO term, while the red line shows the −log_10_ of the FDR. **D.** Heatmap of the gene expression data obtained from the Expression Atlas (https://www.ebi.ac.uk/gxa/home) of the differentially represented interactome proteins. Heatmap was prepared with *Morpheus*, and colors indicate the row minimum (blue) and maximum (red) values. The map shows how half of the proteins is highly expressed in skeletal muscle tissue, while the other half is expressed at various levels across multiple tissues. The names of four proteins decreasing in MT compared to MB are boxed. **E.** Bar graph showing peptide counts of  FXR1 and RBM28 across the four groups (HC MB, HC MT, DMD MB, and DMD MT). **F.** Bar graph showing RNA-seq normalized read counts for *FXR1* and *RBM28* across the MB and MT samples. Error bars in E and F represent standard errors.

To understand whether RBPs could actively participate in the muscle formation process by regulating or affecting the observed shift in gene expression needed to convert MB into MT, we studied whether any RBP was differentially represented in the interactome dataset. We found that 32 RBPs were differentially represented in MT compared to MB, with 28 proteins enriched in MT and 4 proteins enriched in MB (Figure 5A and B; Table 1). Analysis of intensity-based absolute-protein-quantification (iBAQ) data confirmed changes in the same direction as spectral data for all proteins and showed significant differences for 15 RBPs (Figure S3). Most of the proteins showed RNA and nucleotide binding activities (Figure 5C). To better understand the specificity of the identified signature, we searched the gene expression atlas for tissues in which these proteins are preferentially expressed. Roughly half showed prominent muscle expression, while the rest were expressed in multiple tissues at variable levels (Figure 5D). Examples of differentially represented RBPs are FXR1 and RBM28; while FXR1 binding to target transcript was increased upon muscle differentiation, RBM28 binding capacity was shown to be reduced after differentiation. To understand whether the observed changes were due to differences in gene expression or preferential target engaging, we compared interactome data and gene expression data for these two genes. The increased presence of FXR1 in differentiated MT was paralleled by increased *FXR1* expression, while *RBM28* expression was stably low in both MB and MT (Figure 5E and F). To understand whether the increase of RBM28 binding to mRNA targets in MB was caused by an increased availability of target transcripts, we mapped all RBM28 and FXR1 binding sites in the genome (based on the known binding motifs obtained from the CISBP-RNA database http://cisbp-rna.ccbr.utoronto.ca/index.php) and quantified the expression levels of the binding motifs in the available RNA-seq samples. For FXR1, there were indeed more binding motifs present in genes expressed in MT; however, there was no difference in the total number of RBM28 binding motifs across the four conditions that would explain the reduction of RBM28 in the interactome of MT compared to MB (Figure S4), suggesting preferential mRNA target engaging by RBM28 in MB compared to MT. No statistically significant differences were found in the comparison of RBPs between HCs and DMD patients. However, certain RBPs showed interesting trends such as AHNAK (Figure S5), previously reported to play a role in myogenic differentiation [19].

**Table 1:** **RBPs present differentially between MB and MT interactomes**

*Note*: RBP, RNA binding protein; FC, fold change.

### Overlap with interactomes of other cell lines shows common and cell type-specific RBPs

**Figure 6:**
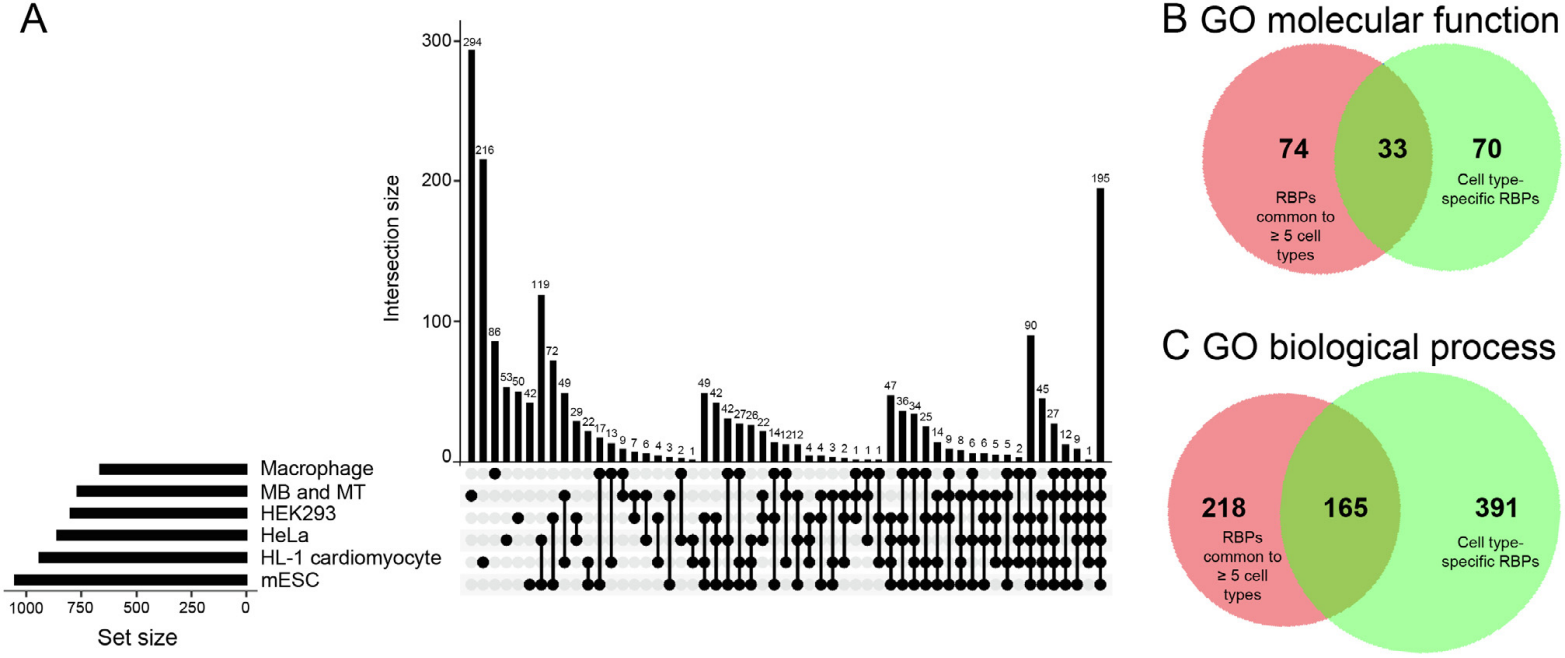
**Identification of common and cell type-specific RBPs** **A.** Plot showing the intersection of interactomes across different cell lines. Horizontal bars on the left show the size (number of proteins) identified in each study. Vertical bars show the number of proteins that are uniquely present in each cell line or in combination of cell lines. The six bars on the leftmost side represent proteins present in unique cell lines, while bars right to them show proteins present in two or more conditions with the last bar on the right showing 195 RBPs that are present in all cell lines and represent a core set of RBPs needed by all cell types but not specific for individual lineages. Muscle cell lines (MB and MT) show the highest number of proteins uniquely present per cell line. The panel below indicates what cell line or combination of cell lines is represented by the corresponding bar on top. **B.** Venn diagrams showing the overlap in molecular functions for RBPs found across five or more cell types and RBPs uniquely identified in specific cell types. **C.** Venn diagrams showing the overlap in biological processes for RBPs found across five or more cell types and RBPs uniquely identified in specific cell types. mESC, mouse embryonic stem cell.

To assess the specificity of the identified interactome, we investigated the overlap of proteins identified in MB and MT with five previously published interactomes in macrophages, HeLa cells, HEK293 cells, mouse HL-1 cardiomyocytes, and mouse embryonic stem cells (mESCs) [12], [14], [15], [16], [17]. A core of 195 proteins was shared across all six cell types, representing a repertoire of conserved RBPs needed to exert basic functions across cells and tissues. A variable number of proteins was shared by groups of two to five cell types. We identified a set of 294 RBPs specific for muscle tissue of which only 19 were reported in a previous census of all RBPs [20] (Figure 6A; Table S8). To understand whether differences exist between cell type-specific RBPs compared to common RBPs, we tested what molecular functions and biological processes are enriched for RBPs uniquely identified in specific cell types compared to RBPs found in five or more cell types (Table S9). Among the significant GO molecular function terms we found 33 shared between unique and common RBPs (*e.g.*, RNA binding, nucleic acid binding, and heterocyclic compound binding), 74 specific for the common RBPs (*e.g.*, single-stranded RNA binding, mRNA 3′-UTR binding, and RNA helicase activity), and 70 specific for RBPs found in unique cell types (*e.g.*, nucleoside phosphate binding, catalytic activity, actin filament binding, and ribonuclease activity) (Figure 6B). A large group of 556 biological processes was enriched for RBPs found in unique cell types and included terms such as RNA phosphodiester bond hydrolysis, regulation of ATPase activity, termination of DNA-templated transcription, and regulation of actin filament depolymerization (Figure 6C).

**Figure 7:**
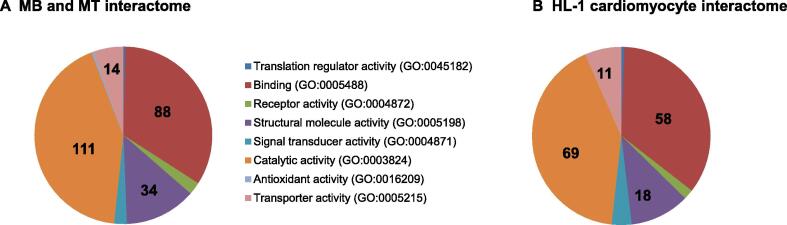
**Direct annotation comparison of the MB and MT specific proteins with the HL-1 cardiomyocytespecific proteins** **A.** Pie chart showing the RBPs uniquely found in the MB and MT interactome (294 RBPs mapped to 307 genes and 261 total function hits). **B.** Pie chart showing RBPs uniquely found in the HL-1 cardiomyocyte interactome (216 RBPs mapped to 221 genes and 166 total function hits). While the proteins are uniquely found in the two experiments and represent condition-specific RBPs, the annotations are highly similar. Numbers in the pie charts represent the number of proteins mapped to each GO entry.

Interestingly, the interactome of skeletal muscle cells was more closely related to mouse HL-1 cardiomyocytes with 49 RBPs shared between the two muscle-derived cell lines (Table S10). Both RBP repertoires showed the highest number of unique RBPs (294 for MB and MT cells and216 for HL-1 cardiomyocytes). Direct comparison of the unique set of proteins identified in our experiments and in cardiomyocytes revealed that skeletal muscle cells and cardiomyocytes showed a remarkably similar distribution of protein function even though different proteins were identified (Figure 7). To understand what protein domains may be enriched in the collected interactome data and in the set of 294 muscle RBPs, we tested the enrichment of Pfam protein domains and compared it to the comprehensive list published by Gerstberger and colleagues [20]. The analysis showed a significant enrichment of 68 Pfam domains in our dataset (Table S11) compared to the 189 Pfam domains mapped to the proteins listed by Gerstberger et al. (Table S12). Forty Pfam domains were found in both lists showing that the majority of RBPs in this study have classical RNA binding domains, while the remaining 28 Pfam domains were previously identified and included intermediate filament protein, TCP-1/cpn60 chaperonin family, Hsp70 protein, and myosin head, among others.

Among the proteins exclusively shared by MB and MT cells and HL-1 cardiomyocytes, we identified STAT3 as an interesting hit, as we recently reported an increased concentration of this protein in circulation in DMD patients [21] (Figure S6). STAT3 was also recently shown to affect the pathophysiology in the mouse model for DMD [22].

## Discussion

Muscle formation is a coordinated process requiring extensive changes in gene expression driving the fusion of multiple cells into multinucleated muscle fibers. While the gene expression signature resulting in the synthesis of myofibrillar proteins involved in the contractile sarcomeres and driving this physiologic process is known, there is no data on the repertoire of RBPs involved in this transition. It has been widely described how RBPs are needed for mRNA localization, turnover, and translation [4], [5], [6], [7], [8]. In this work we studied the repertoire of RBPs in proliferating and differentiated muscle cells in healthy and dystrophic conditions, where the gene expression signature is heavily affected and muscle regeneration and new muscle fiber formation are impaired [23], [24], [25]. Our work shows the enormous gene expression changes upon muscle differentiation in both healthy and dystrophic conditions. A total of 769 RBPs were identified across proliferating and differentiated muscle fibers obtained from both HC- and DMD-derived cells, showing large enrichment for RBPs.

A core of 195 proteins was shared with another five reported interactome datasets spanning from macrophages to ESCs. These RBPs are required to maintain primary metabolic processes such as nonsense codon surveillance (*e.g.*, UPF1), splicing (*e.g.*, NONO), and RNA metabolism (*e.g.*, XRN1). A total of 741 proteins was found in unique cell types. Comparison of RBPs found in unique cell types and RBPs in five or more cell types showed that cell type-specific RBPs are involved in key functions such as RNA metabolic process as well as other less obvious functions such as actin filament depolymerization. These associations may clarify the role of previously unsuspected RBPs in processes such as mRNA trafficking. It has been shown that the cytoplasmic accumulation of human antigen R (HuR), an RBP known to play a role in stabilization and translation of AU-rich elements, is a process guided by actin and myosin [26].

We observed a large fraction of muscle-specific proteins composed of 294 new hits and a pool of 49 proteins shared with the previously reported HL-1 cardiomyocytes [17]. Interestingly, the repertoire of newly identified RBPs shows an almost identical protein class composition to the RBPs uniquely identified in cardiomyocytes, suggesting that different RBPs with similar function are responsible for muscle-specific physiological tasks in skeletal muscle fibers and cardiomyocytes. Of note, this group of unique muscle-specific proteins was enriched with proteins known to possess catalytic activity. While this is somewhat unexpected, a similar result was obtained in cardiomyocytes by Liao and colleagues [17], who hypothesized that these proteins could have a second role as RNA regulators, as previously shown for mitochondrial proteins [27]. While this may turn out to be the true interpretation, we think that capturing with oligo-dT beads could lead to purification of proteins involved in ATP synthesis, which is one of the major pathways in muscle tissue. Among the set of 49 shared proteins between skeletal and cardiac muscles, we identified STAT3, which we recently reported to be a candidate diagnostic biomarker for DMD as its serum concentration is elevated in DMD patients compared to HCs [21]. This finding shows the potential of interactome studies to not only uncover the background biology supporting cell-specific RNA mechanisms, but also to identify clinically relevant tissue-specific biomarkers.

We observed that the concentration of 32 RBPs was affected during myogenesis. Half of these proteins were muscle-specific according to the gene expression atlas (Figure 5). We focused on two proteins, FXR1 and RBM28, which showed opposite directional changes during differentiation. FXR1 was found to be more prevalently bound to mRNAs in differentiated MTs compared to proliferating MBs. *Fxr1* knock-out (KO) mice died at the neonatal stage; whereas mice with reduced FXR1 expression survived longer than KO mice, it showed reduced limb musculature compared to WT mice, suggesting an important role of FXR1 during muscle formation [28]. The levels of FXR1 in the interactome fraction were mirrored by the gene expression levels, supporting a correlation between the protein levels in the cell and the loading of the protein on its target RNA motif. We observed a completely opposite situation for RBM28, which was reduced during differentiation even though transcriptional levels were lower but constant during myogenesis. A motif search for both FXR1 and RBM28 showed how the transcriptome of muscle cells is enriched for FXR1 binding sites compared to RBM28, further supporting the role of FXR1 in myogenesis compared to RBM28. However, the motif expression levels did not clarify the increased motif engaging by RBM28 in MBs.

Our analysis did not allow us to identify RBPs differentially present between healthy and dystrophic cells. It is possible that further studies including more cases and several differentiation time points could identify RBPs that differ between the two situations. Nevertheless, it was encouraging to see a few proteins with interesting trends, such as an increased representation in dystrophic conditions of AHNAK, known to be highly expressed in muscle tissue and involved in muscle regeneration [29], [30].

In conclusion, our study has increased the catalog of known RBPs and allowed us to identify muscle-specific RBPs that play a role in muscle formation. Our data show how we can advance our knowledge of muscle formation regulation as well as lead towards identifying candidate molecules that can be used to develop cell-specific assays with diagnostic potential.

## Materials and methods

### Cell culture

Four distinct immortalized MB cell lines were used for the experiments. Control cell lines were obtained from two healthy individuals (KM155 and AB117). The cell lines 8036 and 6311 were obtained from DMD patients. Cell line 8036 was established from a DMD patient carrying an out-of-frame deletion of exons 48–50, while cell line 6311 carries a deletion of exons 45–50. Both DMD patient lines were the kind gift of Dr. Vincent Mouly (Institute of Myology, Paris, France). MB cells were kept in proliferating medium, while multinucleated MT cells were obtained by serum deprivation of MB cultured to a confluence of 80%, as previously described [31]. Cells were incubated in differentiation medium for 4–5 days. To obtain a sufficient number of cells, we coated 24 Petri dishes (Ø = 15 cm) with 0.5% gelatin and used six dishes per condition (four conditions: MB noCL, MT noCL, MB CL, and MT CL).

### Crosslinking and interactome capture

To crosslink RNA and proteins, we followed the procedure as previously described [13]. Briefly, cells were washed twice with PBS and crosslinked with 0.15 J/cm^2^ using the Stratalinker UV Crosslinker 2400 (Stratagene, La Jolla, CA). Cells from one dish were lysed using 2.8 ml of lysis buffer [20 mM Tris-HCl (pH 7.5), 1 mM EDTA, 500 mM LiCl, 0.5% LiDS, 5 mM DTT, and 0.025% NP-40] and they were harvested with a rubber policeman. The lysate was then transferred to the next petri dish to harvest cells from all dishes belonging to the same group. Lysates were collected in a 50 ml tube and homogenized by passing the whole lysate at least 10 times through a 29 G or 27 G needle. Samples were incubated on ice for 10 min and then stored at −80 °C for up to one week.

For each condition 300 µl of oligo-dT magnetic beads (Catalog No. S1419S, New England Biolabs, Ipswich, MA) were used, which were equilibrated at room temperature in lysis buffer according to the manufacturer’s instructions. The equilibrated beads were added to each sample and the samples were incubated for 1 h at 4 °C with gentle rotation. Beads were then placed on a magnet for approximately 15 min to capture RNA–protein complexes until the supernatant was clear. The supernatant was collected in a new 15 ml tube for a second round of capturing and kept at 4 °C. The beads were resuspended in 3 ml of lysis buffer, split into two 1.5 ml tubes, and incubated for 5 min on ice with consistent agitation (inverted manually ~ 10 times per min). The tubes were placed on a magnet and the supernatant was removed. 1 ml of buffer 1 [20 mM Tris-HCl (pH 7.5), 1 mM EDTA, 500 mM LiCl, 0.1% LiDS, 5 mM DTT, and 0.025% NP-40] was added to each tube, the pellet was resuspended, and the samples were incubated for 5 min on ice with consistent agitation. The supernatant was removed after putting the samples on the magnet. This washing step was repeated once more with buffer 1, then twice with buffer 2 [20 mM Tris-HCl (pH 7.5), 1 mM EDTA, 500 mM LiCl, 5 mM DTT, and 0.025% NP-40] and twice with buffer 3 [20 mM Tris-HCl (pH 7.5), 1 mM EDTA, 200 mM LiCl, and 5 mM DTT]. After washing, captured samples were eluted in a total volume of 200 μl elution buffer [20 mM Tris-HCl (pH 7.5) and 1 mM EDTA] by pooling both samples together. The samples were incubated for 5 min at 55 °C. Immediately, the tubes were put on the magnet and the supernatant containing the RNA–protein complexes was transferred into a new tube. For the second round of capture, the previously collected supernatant was treated with equilibrated oligo-dT beads and the whole procedure was repeated. Samples were then frozen at −80 °C pending use.

### RNA purification and analysis

For RNA analysis, 40 µl of sample were used. The captured RNA–protein complexes were treated with 2 µg proteinase K and 10 µl of 5× proteinase K buffer [50 mM Tris-HCl (pH 7.5), 750 mM NaCl, 1% SDS, 50 mM EDTA, 2.5 mM DTT, and 25 mM CaCl_2_]. Samples were incubated for 1 h at 50 °C. RNA was then purified using the RNeasy Mini Kit (Catalog No. 74104, Qiagen, Hilden, Germany) according to manufacturer’s instructions. Samples were eluted in 40 µl of RNase-free water, and RNA concentrations were measured by NanoDrop spectrophotometer (Catalog No. ND 1000, ThermoFisher Scientific, Waltham, MA). Sample preparation for RNA-seq was performed using the standard poly-A mRNA library preparation of the TruSeq RNA Library Prep Kit v2 (Illumina, San Diego, CA). RNA-seq analysis was performed for MB and MT samples of both KM155 and 8036 cells (total *n* = 4).

### Protein purification

For protein analysis, samples were purified using Amicon ultra centrifugal filters (3 kDa; catalog No. UFC500396, Merck Millipore, Burlington, MA) according to the manufacturer’s instructions. The samples were defrosted and loaded onto the filters, and the tubes were centrifuged at 18,000 *g* for 20 min at 4 °C. 400 μl of buffer 4 [10 mM Tris-HCl (pH 7.5) and 50 mM NaCl] was added to sample in the filter and the centrifugation step was repeated. After removing the supernatant, the filter was placed bottom up in a new collection tube and the remaining solution was spun down for 1 min at 18,000 *g* at 4 °C. The recovered sample volumes of different conditions were determined and adjusted to the highest volume by adding buffer 4. RNA digestion was performed by adding an RNase mix consisting of 0.11 μl RNase buffer [100 mM Tris-HCl (pH 7.5), 1.5 mM NaCl, 0.5% NP-40, and 5 mM DTT], 0.04 U RNase A (Catalog No. 19101, Qiagen) and 0.04 U RNase T1 (Catalog No. EN0541, ThermoFisher Scientific) per μl of sample and incubating for 90 min at 37 °C followed by 15 min at 50 °C with gentle agitation.

### SDS-PAGE and silver staining

The obtained protein samples were loaded onto a Criterion XT 4%–12% BisTris precast gel (Catalog No. 3450123, BioRad, Hercules, CA) in a Biorad chamber filled with 1× XT MOPS Running Buffer (Catalog No. 161-0788, BioRad). A total of 12 µl of sample with 4 µl 4× NuPage sample buffer (Catalog No. NP0007, Life Technologies, Carlsbad, CA) were first denatured for 5 min at 95 °C and then loaded onto the gel together with 5 µl of protein marker (PageRuler; catalog No. 26619, ThermoFisher Scientific). The gel was run for 45 min at 200 V followed by an incubation in fixing solution (40% ethanol, 10% acetic acid, and 50% H_2_O) for 1 h. The gel was washed with water for at least 30 min before sensitization with 0.02% sodium thiosulfate solution for 1 min. The gel was washed three times for 20 s with water and was incubated for 20 min at 4 °C in 0.1% silver nitrate solution. Residual silver nitrate was removed by washing the gel three times for 20 s with water. Before imaging the gel was developed with a 3% sodium carbonate / 0.05% formaldehyde solution until the staining was sufficient. The gel was washed again for 20 s with water and left in 5% acetic acid for 5 min to terminate the staining.

### Protein analysis

Samples were incubated at 90 °C with LDS sample buffer (Life Technologies). Proteins were separated on a 4%–12% PAGE (NuPAGE Bis-Tris Precast Gel, Life Technologies) after a run for 1 cm. Staining was performed with silver (SilverQuest Silver Stain, Life Technologies). Proteomic analysis was performed as previously described [32]. The lane was cut in three parts and subjected to reduction by DTT followed by alkylation by iodoacetamide and in-gel digestion with trypsin with the Proteineer DP digestion robot (Bruker, Billerica, MA). Tryptic peptides were lyophilized, dissolved in 95/3/0.1 (v/v/v) water/acetonitril/formic acid, and analyzed by on-line C18 nanoHPLC MS/MS with an Easy nLC 1000 gradient HPLC system (Thermo, Bremen, Germany) and LUMOS mass spectrometer (Thermo). Peak lists were obtained from raw data and submitted to the *Homo sapiens* database at Uniprot, using Mascot v. 2.2 for protein identification. Protein with at least two peptides with a threshold of ≥95% were sorted and compared using Scaffold software version 4.7.5 (www.proteomesoftware.com).

### Data analysis

The mass spectrometry proteomics data have been deposited to the ProteomeXchange Consortium via the PRIDE [33] partner repository. Differential expression analysis of RNA-seq data was performed on 23,126 genes that showed more than ten counts across the four samples. We tested for differences in cell type (MB *vs*. MT) and diagnosis (HC *vs*. DMD). A Benjamini–Hochberg false discovery rate (FDR) correction for multiple testing was applied. We considered *P* values below 0.05 significant. The GeneTrail2 tool [34] was used to perform overrepresentation analysis for RNA-seq data using GO, Reactome, and WikiPathways as background information. A two-sided test was performed and the Benjamini–Yekutieli multiple testing correction was applied. Bioinformatic analysis to map the Pfam domains was performed using STRING [35]. Protein IDs (Table S7) were compared to the protein IDs provided by Gerstberger and colleagues [20]. Comparison of CL and noCL samples was performed using four samples (two noCL and two CL samples obtained from HC KM155 MB cells). Proteins with common IDs across the replicate experiments were tested. Differential representation was tested using the DeSeq2 package on 262 proteins. Additional data analysis including data from eight samples (HC MB noCL, HC MT noCL, DMD MB noCL, DMD MT noCL, HC MB CL, HC MT CL, DMD MB CL, and DMD MT CL) was performed on a total of 655 proteins. The comparison between CL and noCL samples was in this case performed for cell type (MB and MT) and group (HC and DMD). To compare the interactome across cell types and groups we analyzed samples in triplicate with a total of 12 samples. Samples were either MB or MT from HCs (*n* = 3 each, of which two replicates of KM155 cells and one replicate of AB117 cells), or, MB or MT of DMD patients (*n* = 3 each, two replicates of 8036 cells and one replicate of 6311 cells). To analyze the data we started with the more complex and comprehensive model including both cell type (MB or MT) and diagnosis (HC or DMD) as covariates and proceeded to simplify the model by testing only the difference between MB and MT as contribution of diagnosis was not significant. Benjamini–Hochberg FDR correction for multiple testing was applied and *P* values below 0.05 were considered significant. Analysis of protein data was performed on 12 CL samples. We filtered out proteins with less than five spectral counts across all samples, resulting in the exclusion of 366 proteins. We tested whether differences existed in the remaining 769 proteins. Confirmation of quantitative differences was performed on the identified RBPs using MaxQuant iBAQ quantitation data analyzed using the *kruskal.test* function in R. Analysis of data was performed in R with DeSeq2 [36], ggplot2 [37], and UpSetR [38] packages.

## Data availability

The mass spectrometry proteomics data have been deposited to the ProteomeXchange Consortium via the PRIDE partner repository (ProteomeXchange: PXD015471), and are publicly accessible at http://www.ebi.ac.uk/pride/archive/projects/PXD015471.

## CRediT author statement

**Monika Hiller:** Methodology, Investigation, Data curation, Writing - review & editing. **Miriam Geissler:** Methodology, Investigation, Data curation, Writing - review & editing. **George Janssen:** Methodology, Investigation, Data curation, Writing - review & editing. **Peter van Veelen:** Supervision, Resources, Writing - review & editing. **Annemieke Aartsma-Rus:** Supervision, Resources, Writing - review & editing. **Pietro Spitali:** Conceptualization, Formal analysis, Writing - original draft, Visualization, Supervision, Resources, Project administration, Funding acquisition. All authors read and approved the final manuscript.

## Competing interests

Authors declare no conflict of interest related to this work.

## Supplementary data

Supplementary Figure S1 **Comparison of CL and noCL samples. A.** Silver staining of an SDS-PAGE gel showing noCL and CL samples. L, molecular ladder. **B.** Volcano plot showing the enrichment of proteins in four CL samples compared to four noCL ones. A total of 655 proteins were tested and a significant enrichment was found for 124 proteins (adjusted *P* < 0.1). The log2 FC in CL vs. noCL samples is plotted on the *x*-axis and the −log10 of the *P* value is plotted on *y*-axis. Red dots represent proteins showing a significant FC in CL samples compared to noCL ones. The eight samples included in this analysis are HC MB noCL, HC MT noCL, DMD MB noCL, DMD MT noCL, HC MB CL, HC MT CL, DMD MB CL, and DMD MT CL. **C.** Bar graph showing the increase of known RBPs after crosslinking. **D.** GO analysis showing molecular function terms for the 124 proteins significantly enriched in CL samples. Black bars represent the total number of counted proteins (belonging to the 124 significant ones) per GO category. The red line shows the −log10 of the FDR. The boundaries shown on the *y*-axis refer to both bar values and line values.

Supplementary Figure S2 **GO classification analysis of HC MB, HC MT, DMD MB, and DMD MT cells** Panels **A**, **D**, **G**, and J show the classification of GO molecular function terms in all four groups. These panels show that the majority of proteins have binding activity (red). The subgroup of proteins with binding activity is considered in the middle panels (**B**, **E**, **H**, and **K**), where proteins are classified based on the type of binding. Enrichment for proteins binding nucleic acids is shown (blue). The subgroup of proteins with nucleic acid binding activity is further considered in right panels (**C**, **F**, **I**, and **L**), which show that most of the nucleic acid binding proteins are RNA binding proteins (blue).

Supplementary Figure S3 **Analysis of iBAQ intensities for the RBPs differentially represented between MB and MT**. **A.** iBAQ intensities of GAPDH and ELAVL2/4, which show much higher levels compared to other RBPs. **B.** Other RBPs quantified by iBAQ intensity. Boxed names highlight significant differences (*P* < 0.05) between MB and MT tested using the Kruskal–Wallis test.

Supplementary Figure S4 **Motif analysis for FXR1 and RBM28. A.** Graph showing the distribution of motifs recognized by FXR1 and RBM28 across the genome. **B.** Bar graph showing the motif expression levels for both FXR1 and RBM28 motifs across the four groups. The expression levels were obtained by multiplying the total number of binding sites for each gene for the normalized gene expression levels. In both panels, FXR1 is plotted in dark gray and RBM28 in light gray.

Supplementary Figure S5 **Two exemplar RBPs showing a trend toward an increase in the interactome of cells derived from DMD patients compared to HCs.**

Supplementary Figure S6 **Increased STAT3 serum levels in DMD patients (*n* = 15) compared to HCs (*n* = 9) by linear regression (adjusted *P* < 0.01).**

Supplementary Table S1 **Count data of CL and noCL cells.**

Supplementary Table S2 **GO analysis of RNA-seq data.**

Supplementary Table S3 **Reactome pathways of RNA-seq data.**

Supplementary Table S4 **WikiPathways of RNA-seq data.**

Supplementary Table S5 **Gene expression results of MB and MT.**

Supplementary Table S6 **Gene expression results of cells derived from DMD patients and HCs.**

Supplementary Table S7 **Count table of mass spectrometry interactome data.**

Supplementary Table S8 **List of 294 RBPs specific for muscle tissue.**

Supplementary Table S9 **GO analysis of unique and common RBPs.**

Supplementary Table S10 **RBPs in common between muscle cells and HL-1 cardiomyocytes.**

Supplementary Table S11 **Pfam domains enriched in MB and MT cells.**

Supplementary Table S12 **Pfam domains extracted from literature.**
