# Supplementary material for: The mRNA Binding Proteome of Proliferating and Differentiated Muscle Cells
Source: Genomics Proteomics Bioinformatics. 2020 Dec 16;18(4):384–96. doi: 10.1016/j.gpb.2020.06.004 (PMC8242265; doi:10.1016/j.gpb.2020.06.004)
Supplement: Supplementary Figure S1 — Comparison of CL and noCL samples. A. Silver staining of an SDS-PAGE gel showing noCL and CL samples. L, molecular ladder. B. Volcano plot showing the enrichment of proteins in four CL samples compared to four noCL ones. A total of 655 proteins were tested and a significant enrichment was found for 124 proteins (adjusted P < 0.1). The log2 FC in CL vs. noCL samples is plotted on the x-axis and the −log10 of the P value is plotted on y-axis. Red dots represent proteins showing a significant FC in CL samples compared to noCL ones. The eight samples included in this analysis are HC MB noCL, HC MT noCL, DMD MB noCL, DMD MT noCL, HC MB CL, HC MT CL, DMD MB CL, and DMD MT CL. C. Bar graph showing the increase of known RBPs after crosslinking. D. GO analysis showing molecular function terms for the 124 proteins significantly enriched in CL samples. Black bars represent the total number of counted proteins (belonging to the 124 significant ones) per GO category. The red line shows the −log10 of the FDR. The boundaries shown on the y-axis refer to both bar values and line values. [file mmc1.pptx]

## Slide 1
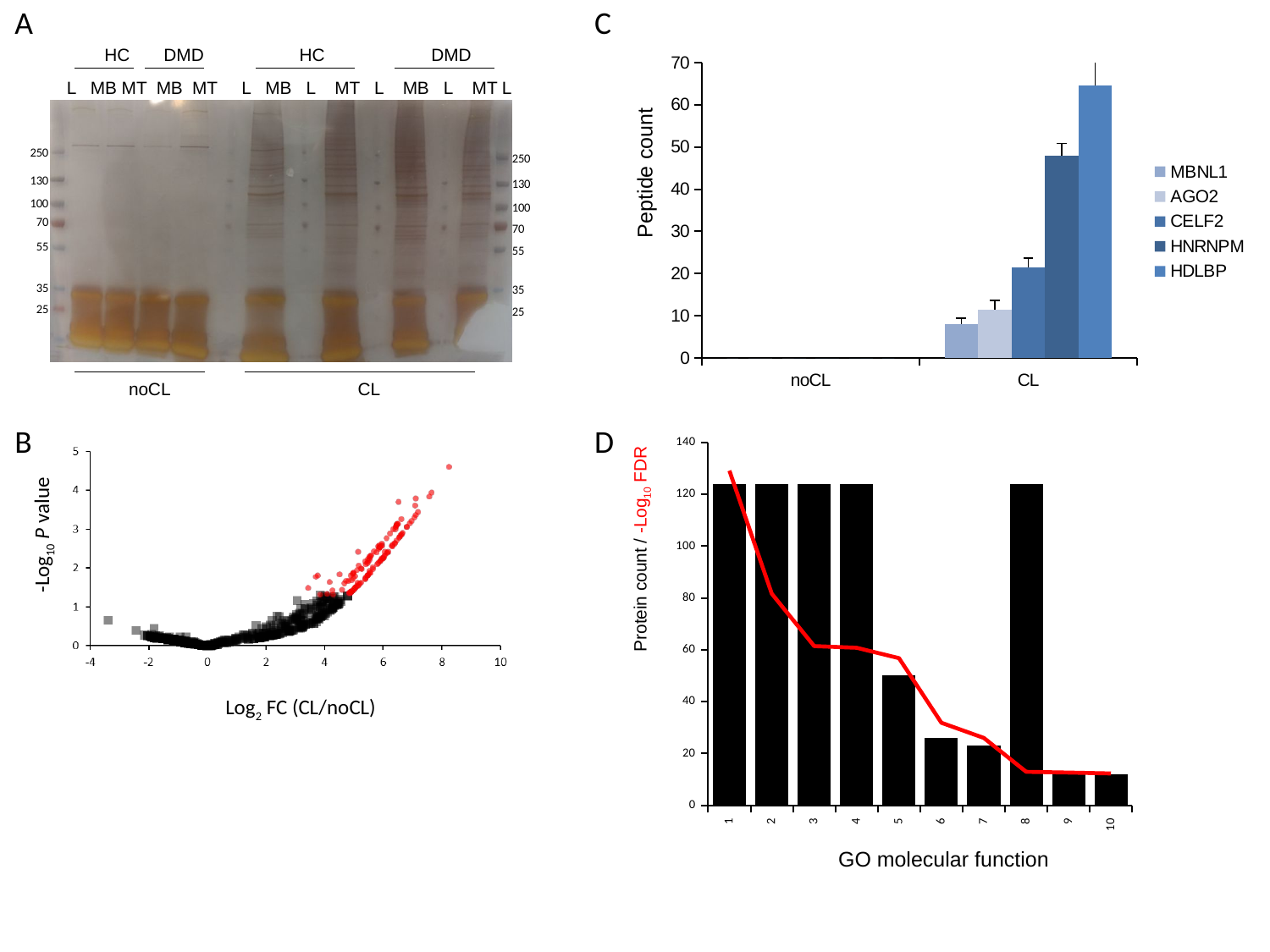

A				 C
B				 D
HC DMD	 HC	 DMD
### Chart
| Category | MBNL1 | AGO2 | CELF2 | HNRNPM | HDLBP |
|---|---|---|---|---|---|
| noCL | 0.0 | 0.0 | 0.0 | 0.0 | 0.0 |
| CL | 8.0 | 11.5 | 21.5 | 48.0 | 64.5 |L MB MT MB MT L MB L MT L MB L MT L
250
250
Peptide count
130
130
100
100
70
70
55
55
35
35
25
25
noCL	 CL
### Chart
| Category | | |
|---|---|---|
-Log10 P value
Protein count / -Log10 FDR
Log2 FC (CL/noCL)
GO molecular function
