# Supplementary figures and images for: The mRNA Binding Proteome of Proliferating and Differentiated Muscle Cells
Source: Genomics Proteomics Bioinformatics. 2020 Dec 16;18(4):384–96. doi: 10.1016/j.gpb.2020.06.004 (PMC8242265; doi:10.1016/j.gpb.2020.06.004)

## Slide 1
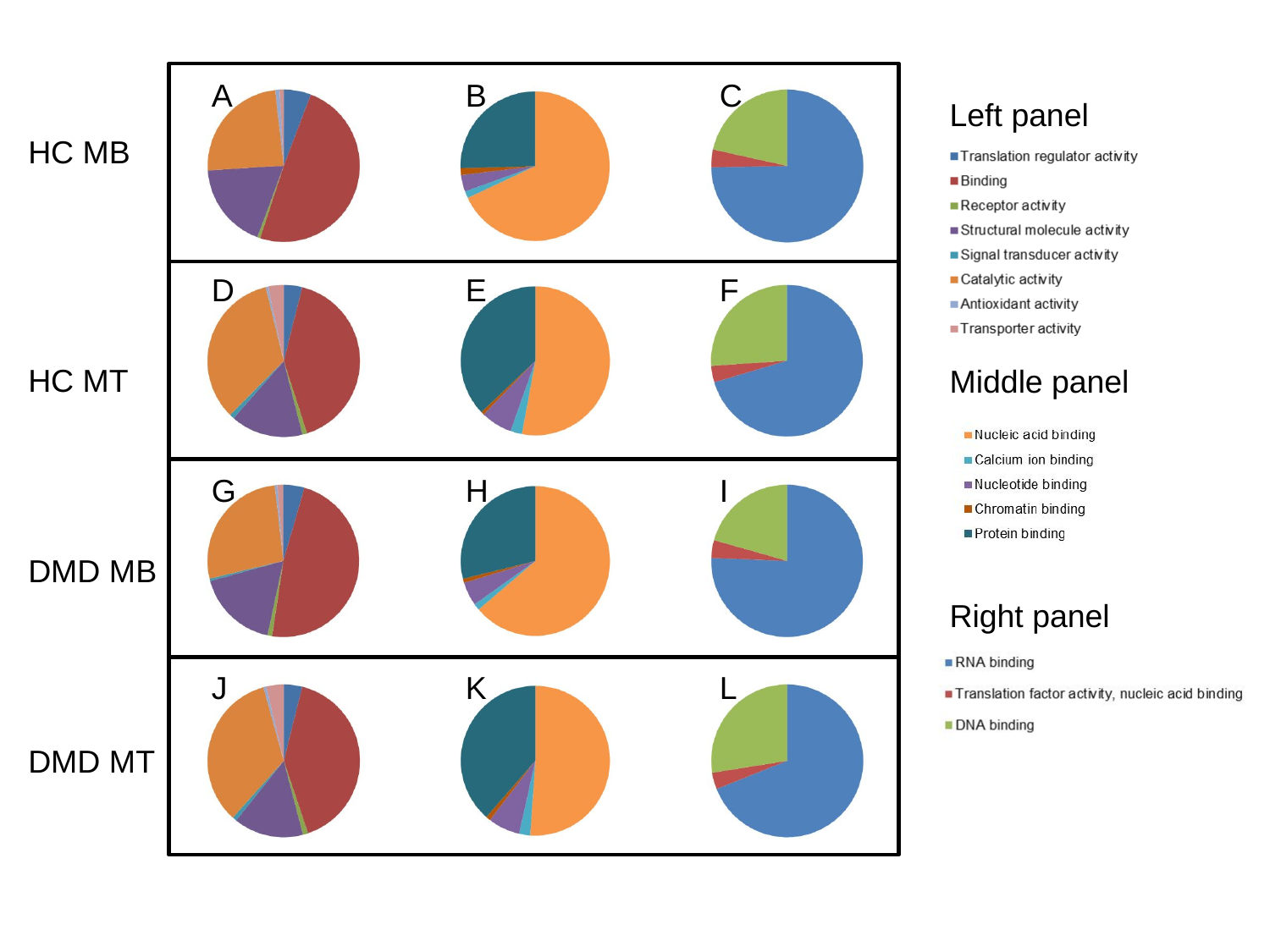

A		B		C
D		E		F
G		H		I
J		K		L
Left panel
Middle panel
Right panel
HC MB
HC MT
DMD MB
DMD MT

Supplement: Supplementary Figure S2 — GO classification analysis of HC MB, HC MT, DMD MB, and DMD MT cells Panels A, D, G, and J show the classification of GO molecular function terms in all four groups. These panels show that the majority of proteins have binding activity (red). The subgroup of proteins with binding activity is considered in the middle panels (B, E, H, and K), where proteins are classified based on the type of binding. Enrichment for proteins binding nucleic acids is shown (blue). The subgroup of proteins with nucleic acid binding activity is further considered in right panels (C, F, I, and L), which show that most of the nucleic acid binding proteins are RNA binding proteins (blue). [file mmc2.pptx]

## Slide 1
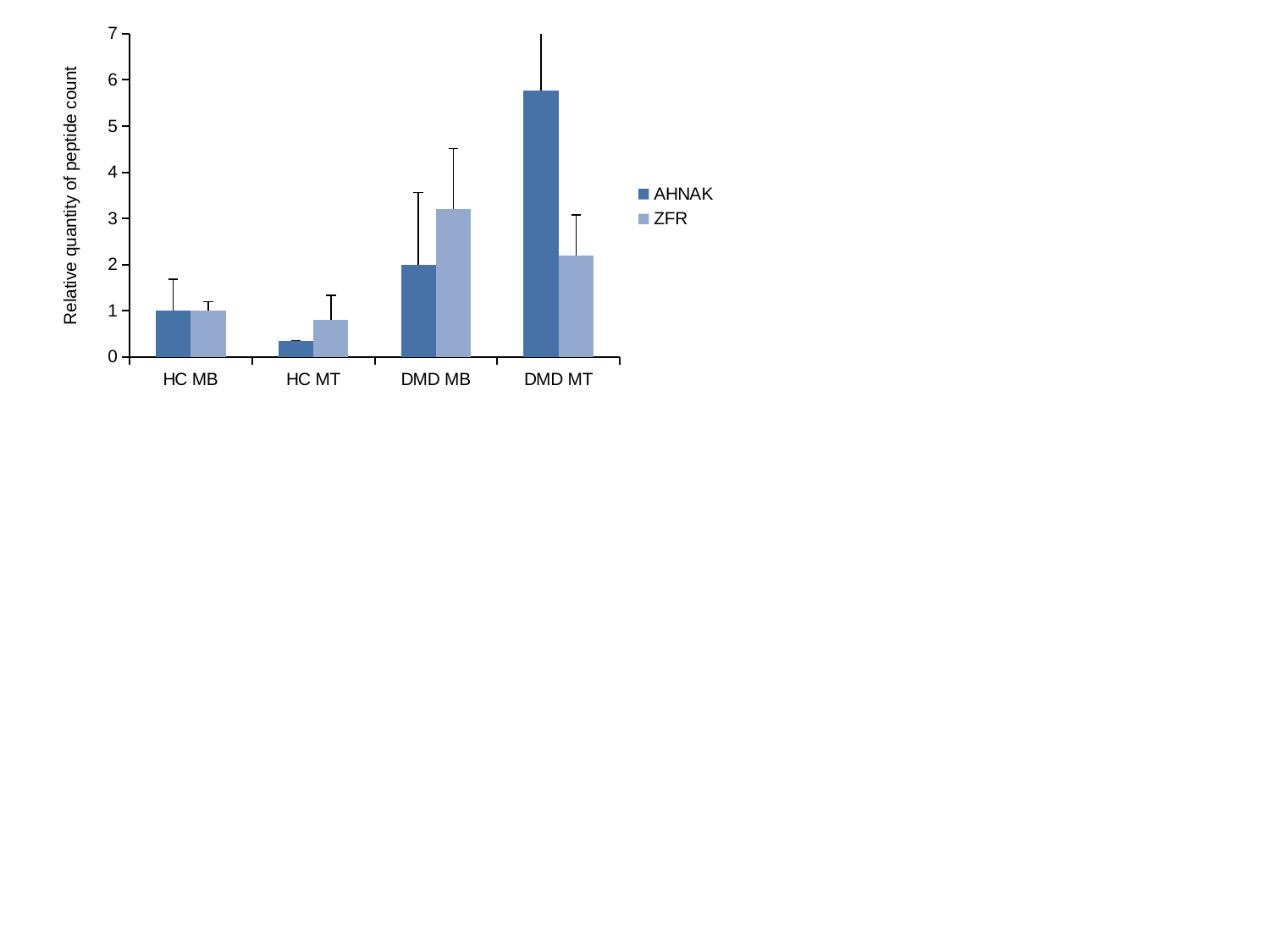

### Chart
| Category | AHNAK | ZFR |
|---|---|---|
| HC MB | 1.0 | 1.0 |
| HC MT | 0.3529411764705882 | 0.7999999999999999 |
| DMD MB | 2.0 | 3.1999999999999997 |
| DMD MT | 5.76470588235294 | 2.1999999999999997 |Relative quantity of peptide count

Supplement: Supplementary Figure S5 — Two exemplar RBPs showing a trend toward an increase in the interactome of cells derived from DMD patients compared to HCs. [file mmc5.pptx]

## Slide 1
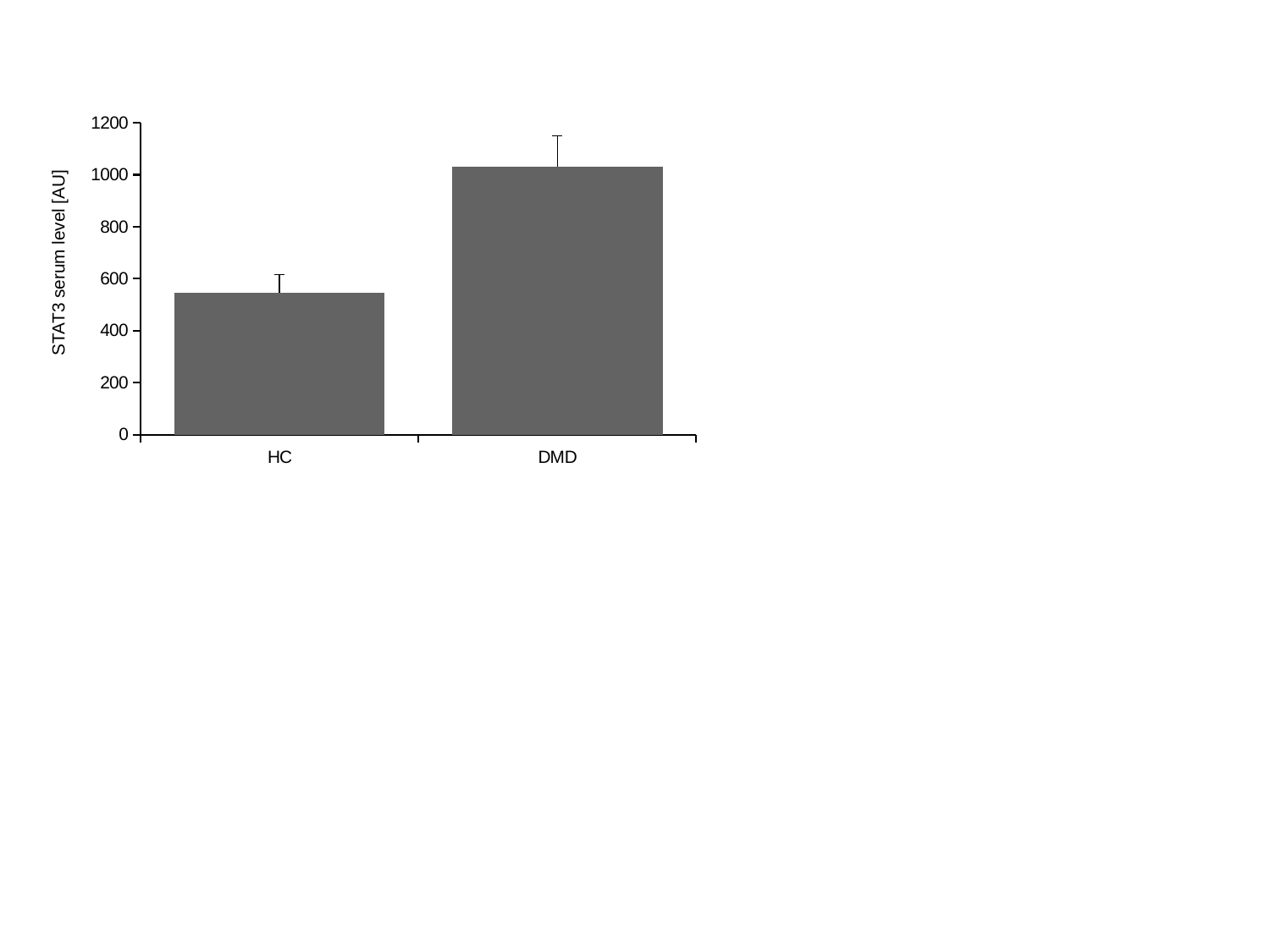

### Chart
| Category | |
|---|---|
| HC | 546.5222222222222 |
| DMD | 1030.24 |STAT3 serum level [AU]

Supplement: Supplementary Figure S6 — Increased STAT3 serum levels in DMD patients (n = 15) compared to HCs (n = 9) by linear regression (adjusted P < 0.01). [file mmc6.pptx]
