# Supplementary material for: The mRNA Binding Proteome of Proliferating and Differentiated Muscle Cells
Source: Genomics Proteomics Bioinformatics. 2020 Dec 16;18(4):384–96. doi: 10.1016/j.gpb.2020.06.004 (PMC8242265; doi:10.1016/j.gpb.2020.06.004)
Supplement: Supplementary Figure S3 — Analysis of iBAQ intensities for the RBPs differentially represented between MB and MT. A. iBAQ intensities of GAPDH and ELAVL2/4, which show much higher levels compared to other RBPs. B. Other RBPs quantified by iBAQ intensity. Boxed names highlight significant differences (P < 0.05) between MB and MT tested using the Kruskal–Wallis test. [file mmc3.pptx]

## Slide 1
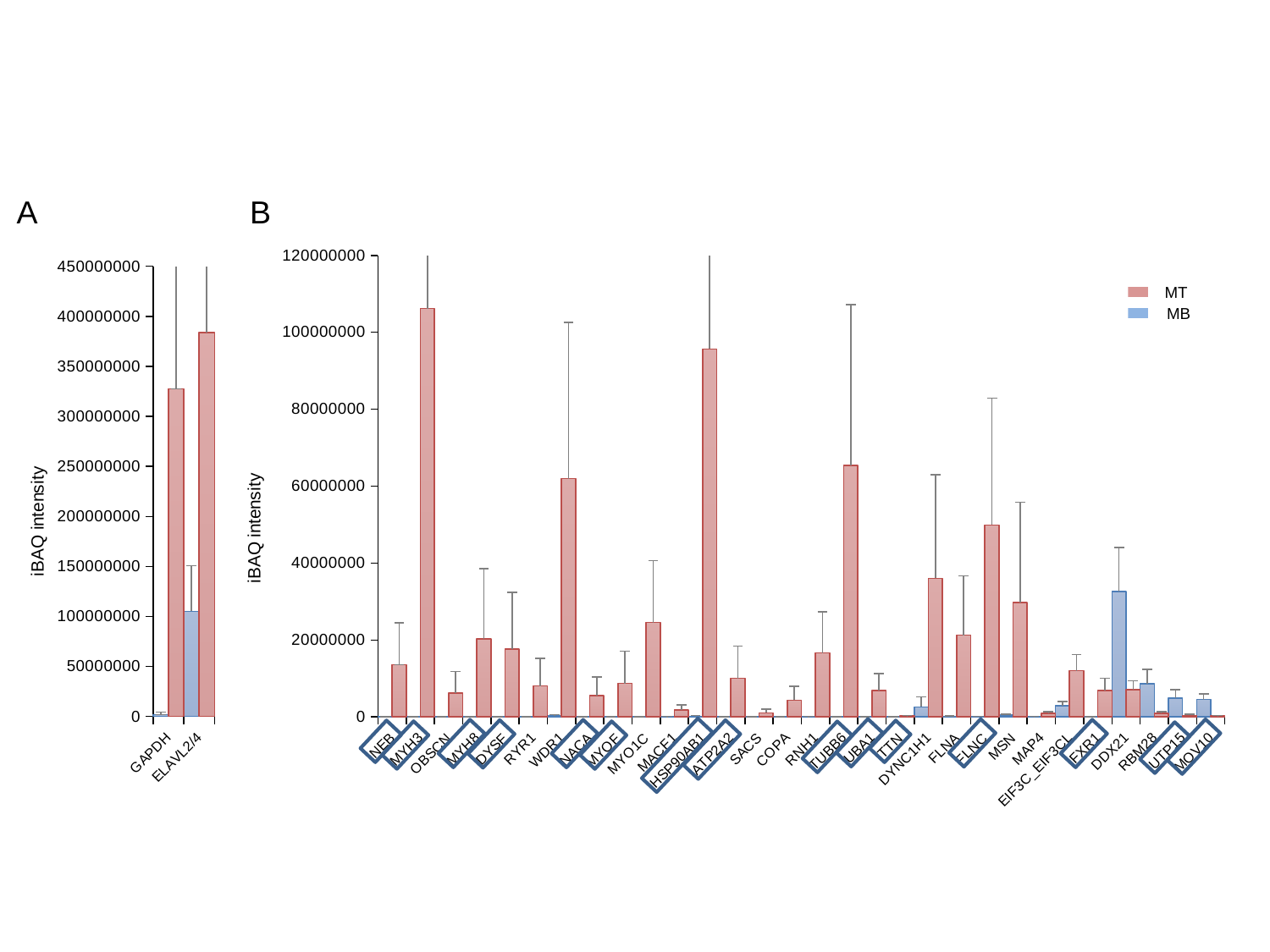

A	 B
### Chart
| Category | MB | MT |
|---|---|---|
| NEB | 13996.5 | 13609413.333333334 |
| MYH3 | 0.0 | 106156043.66666667 |
| OBSCN | 15069.666666666666 | 6232270.666666667 |
| MYH8 | 0.0 | 20317251.666666668 |
| DYSF | 0.0 | 17635060.333333332 |
| RYR1 | 0.0 | 8046300.0 |
| WDR1 | 278483.3333333333 | 61954696.666666664 |
| NACA | 0.0 | 5578178.333333333 |
| MYOF | 0.0 | 8702044.5 |
| MYO1C | 0.0 | 24528550.0 |
| MACF1 | 91689.83333333333 | 1841018.3333333333 |
| HSP90AB1 | 175869.16666666666 | 95663287.5 |
| ATP2A2 | 0.0 | 10047623.5 |
| SACS | 0.0 | 1077858.3333333333 |
| COPA | 27981.666666666668 | 4343589.666666667 |
| RNH1 | 69240.0 | 16623681.666666666 |
| TUBB6 | 20613.333333333332 | 65424183.333333336 |
| UBA1 | 0.0 | 6819061.166666667 |
| TTN | 0.0 | 208012.66666666666 |
| DYNC1H1 | 2613210.5 | 36044880.0 |
| FLNA | 173716.0 | 21262588.5 |
| FLNC | 109075.5 | 49931369.5 |
| MSN | 430449.6666666667 | 29721933.333333332 |
| MAP4 | 88970.33333333333 | 872520.8333333334 |
| EIF3C_EIF3CL | 2898892.8333333335 | 12014006.666666666 |
| FXR1 | 0.0 | 6809960.833333333 |
| DDX21 | 32648816.666666668 | 7035700.0 |
| RBM28 | 8668723.333333334 | 952491.6666666666 |
| UTP15 | 4946655.0 | 416781.6666666667 |
| MOV10 | 4524695.0 | 275624.3333333333 |
### Chart
| Category | MB | MT |
|---|---|---|
| GAPDH | 1665996.6666666667 | 327728401.6666667 |
| ELAVL2/4 | 105127050.0 | 383670083.3333333 |MT
MB
