# Supplementary material for: The mRNA Binding Proteome of Proliferating and Differentiated Muscle Cells
Source: Genomics Proteomics Bioinformatics. 2020 Dec 16;18(4):384–96. doi: 10.1016/j.gpb.2020.06.004 (PMC8242265; doi:10.1016/j.gpb.2020.06.004)
Supplement: Supplementary Figure S4 — Motif analysis for FXR1 and RBM28. A. Graph showing the distribution of motifs recognized by FXR1 and RBM28 across the genome. B. Bar graph showing the motif expression levels for both FXR1 and RBM28 motifs across the four groups. The expression levels were obtained by multiplying the total number of binding sites for each gene for the normalized gene expression levels. In both panels, FXR1 is plotted in dark gray and RBM28 in light gray. [file mmc4.pptx]

## Slide 1
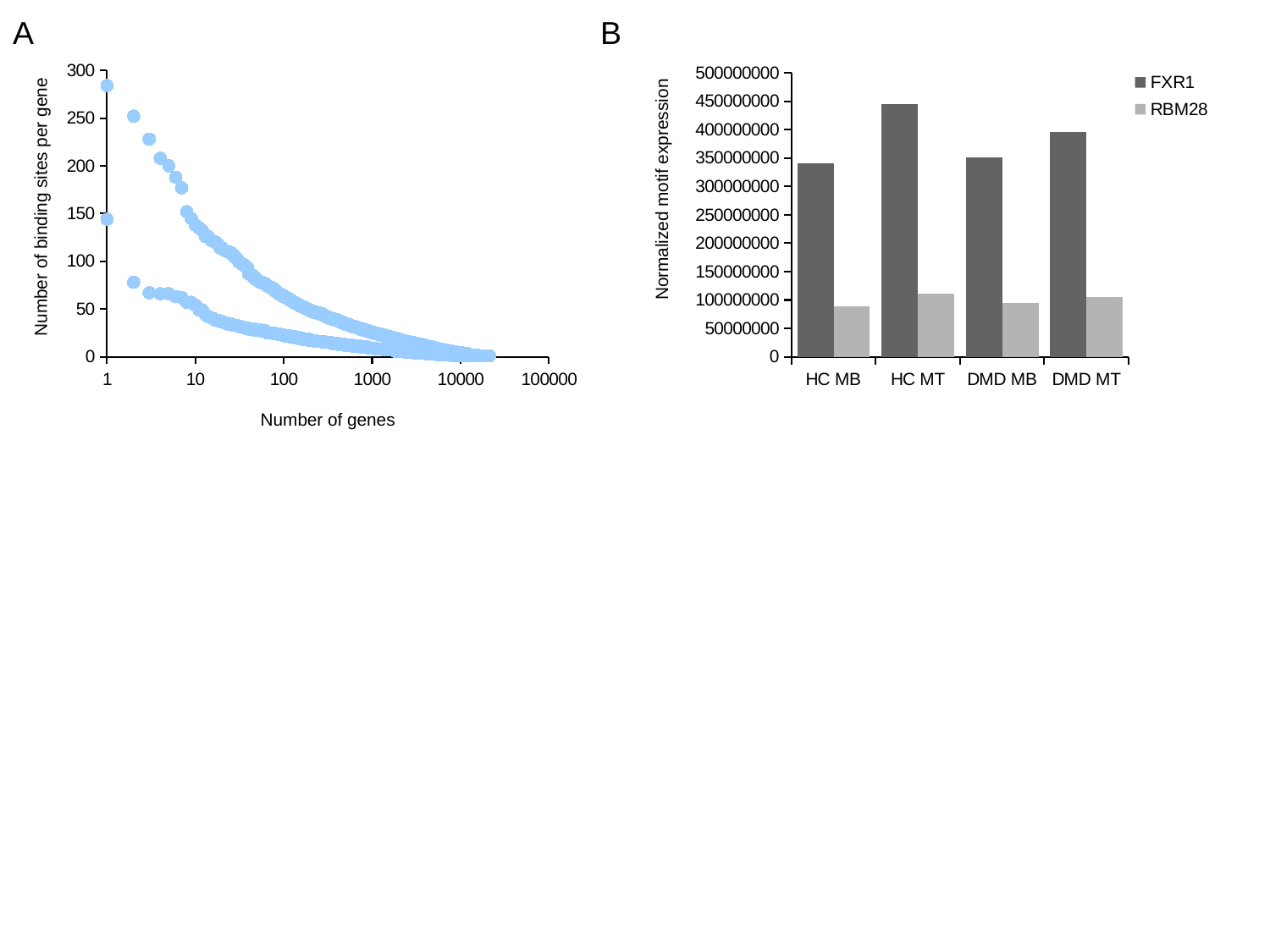

A				 B
### Chart
| Category | | |
|---|---|---|
### Chart
| Category | FXR1 | RBM28 |
|---|---|---|
| HC MB | 340248247.3919955 | 89465409.35241297 |
| HC MT | 444759025.1592533 | 111388098.66312902 |
| DMD MB | 351337914.4052816 | 95397498.05165397 |
| DMD MT | 395436193.39240426 | 105457617.2166772 |Normalized motif expression
Number of binding sites per gene
Number of genes
